# Perspectives Toward Seeking Treatment Among Patients With Psoriasis: Protocol for a Twitter Content Analysis

**DOI:** 10.2196/13731

**Published:** 2021-02-18

**Authors:** Katja Reuter, Delphine Lee

**Affiliations:** 1 Southern California Clinical and Translational Science Institute Keck School of Medicine University of Southern California Los Angeles, CA United States; 2 Department of Public Health and Preventive Medicine SUNY Upstate Medical University Syracuse, NY United States; 3 Division of Dermatology Department of Medicine Harbor-UCLA Medical Center Torrance, CA United States; 4 The Lundquist Institute Torrance, CA United States

**Keywords:** infodemiology, infoveillance, internet, surveillance, patient opinion, psoriasis, treatment, Twitter, social media, social network

## Abstract

**Background:**

Psoriasis is an autoimmune disease estimated to affect more than 6 million adults in the United States. It poses a significant public health problem and contributes to rising health care costs, affecting people’s quality of life and ability to work. Previous research showed that nontreatment and undertreatment of patients with psoriasis remain a significant problem. Perspectives of patients toward seeking psoriasis treatment are understudied. Social media offers a new data source of user-generated content. Researchers suggested that the social network Twitter may serve as a rich avenue for exploring how patients communicate about their health issues.

**Objective:**

The objective of this study is to conduct a content analysis of Twitter posts (in English) published by users in the United States between February 1, 2016, and October 31, 2018, to examine perspectives that potentially influence the treatment decision among patients with psoriasis.

**Methods:**

User-generated Twitter posts that include keywords related to psoriasis will be analyzed using text classifiers to identify themes related to the research questions. We will use Symplur Signals, a health care social media analytics platform, to access the Twitter data. We will use descriptive statistics to analyze the data and identify the most prevalent topics in the Twitter content among people with psoriasis.

**Results:**

This study is supported by the National Center for Advancing Translational Science through a Clinical and Translational Science Award award. Study approval was obtained from the institutional review board at the University of Southern California. Data extraction and cleaning are complete. For the time period from February 1, 2016, to October 31, 2018, we obtained 95,040 Twitter posts containing terms related to “psoriasis” from users in the United States published in English. After removing duplicates, retweets, and non-English tweets, we found that 75.51% (52,301/69,264) of the psoriasis-related posts were sent by commercial or bot-like accounts, while 16,963 posts were noncommercial and will be included in the analysis to assess the patient perspective. Analysis was completed in Summer 2020.

**Conclusions:**

This protocol paper provides a detailed description of a social media research project including the process of data extraction, cleaning, and analysis. It is our goal to contribute to the development of more transparent social media research efforts. Our findings will shed light on whether Twitter provides a promising data source for garnering patient perspective data about psoriasis treatment decisions. The data will also help to determine whether Twitter might serve as a potential outreach platform for raising awareness of psoriasis and treatment options among patients and implementing related health interventions.

**International Registered Report Identifier (IRRID):**

DERR1-10.2196/13731

## Introduction

### Background and Rationale

Psoriasis is an autoimmune disease that causes patches of thick red skin and silvery scales and affects more than 6 million adults in the United States [1,2]. The condition can impact the quality of life and psychological and social functioning [3] and poses a significant public health problem [2,4,5]. A total direct cost of $649.6 million for approximately 1.4 million individuals with clinically significant psoriasis was reported [6], affecting employment and individuals’ ability to work [7]. The condition occurs mostly in adults, men and women alike (ages 18 years and older) but can also affect children and is most common in people aged 50 to 69 years [4].

Previous research showed that nontreatment and undertreatment of patients with psoriasis remain a significant problem in the United States [2,8,9]. Despite several treatment options, 9% to 30% of patients with severe and nearly 50% of patients with mild psoriasis symptoms do not receive treatment, and patients report widespread treatment dissatisfaction [10]. More specifically, up to 30% of patients with severe and nearly 50% of patients with mild psoriasis symptoms do not receive treatment [8]. Known barriers to seeking treatment include a limited understanding of the disease, insurance coverage and out-of-pocket costs, and safety profile concerns [11]. However, perspectives among psoriasis patients toward seeking treatment are understudied. For example, a PubMed search including the terms “psoriasis,” “patients,” and “seeking treatment” results in 4 reports, of which only one identifies treatment-seeking motivations of psoriasis patients [12]. In this study, we define perspective as any expression of thought, viewpoint, or attitude toward health issues and concerns. Efforts that improve the understanding of patients’ perspectives could inform and enhance advocacy and education to ensure that effective treatments are accessible to these patients.

### Social Media and Health Research

Social media includes widely accessible web-based and mobile technologies that allow users to participate in social networking and view, create, and share information online [13]. These communication tools provide a unique source for data mining of health conditions and concerns, serving as a massive focus group of sorts [14-16]; 72% of American adults use at least one social media platform [17].

The emergence of social media has created new sources of analyzable data [11] and led to new research fields (ie, infodemiology and infoveillance) [16,18]. The data social media users generate through their online activities are referred to as their digital footprint [19] or social mediome [20]. Recently, the US Food and Drug Administration (FDA) encouraged the use of unstructured patient-generated health data (PGHD) from different sources including social media to generate insight into patient-experienced outcomes in the real world [21-23]. On Twitter, for example, health surveillance researchers have used these data to gain insight into public perspectives on a variety of diseases and health topics such as influenza, autism, schizophrenia, smoking, HIV/AIDS, and sun-related issues and skin cancer [24-30]. In some cases, social media user data demonstrated a correlation between the disease prevalence and frequency with which Twitter users discussed a disease [31]. The use of PGHD from social media offers a new opportunity to learn about patients’ disease experience and networks that are not otherwise easily captured through traditional surveys or administrative data [32].

### The Social Network Twitter

Nearly 22% of US adults use the social network Twitter including Hispanics (25%), Blacks (24%), and Whites (21%); more than 40% use the platform daily [17]. Twitter users can post short messages (tweets) of up to 280 characters and search for any public message and further engage with these tweets (ie, they can like, reply, and retweet [share] them). Twitter is a primarily public social network; by default, basic Twitter account information such as the profile name, description, and location are public unless a user decides to opt out and make an account private. Due to the more public nature of Twitter, previous research suggested that Twitter provides a “rich and promising avenue for exploring how patients conceptualize and communicate about their specific health issues” [33]. The increasing use of Twitter among members of disease communities is further evidenced by the abundance of disease and health topic hashtags used in the messages [34-36]. A hashtag is a word or phrase preceded by a hash or pound sign (#) and used to identify messages on a specific topic (eg, #psoriasis, #skinchat, #PsoriaticArthritis). These hashtags are used by Twitter users to assign their message to a topic and join ongoing conversations. Users can click on a hashtag and view all of the messages that include the same hashtag and, hence, discuss the same topic. This allows users to form online communities and share their health concerns, disease experience, and questions with like-minded users [37]. However, there is little information about the use of social media among psoriasis patients.

### Previous Work

Few studies have examined social media content about psoriasis. Three studies of YouTube videos showed that misinformation is prevalent on social media and patients are exposed to a wide variety of information, with most of the content being of low quality [38-40]. Another study of dermatology-related content including psoriasis on the photo-sharing social network Instagram demonstrated that information by private offices, cosmetic products, and some patient advocacy groups dominates the user experience, while the use of a large number of hashtags related to dermatological conditions suggests that people use Instagram to post personal experiences with skin conditions [41]. While preparations for this study were underway, Menzies et al [42] published their Twitter analysis of attitudes toward psoriasis treatment among Twitter users. However, there were a few issues with their methodology that weakened their findings [43]. One of the major issues pertained to the fact that the authors did not account for commercial and bot-like content within their dataset. Bots (robots) are purely automated accounts or human-assisted automated accounts (cyborgs) [44-48]. Identifying commercial and bot-like content, which is abundant on social media [44], is critical to discern patients’ perspectives. Furthermore, the authors did not discuss whether and how they controlled for bias introduced by Twitter posts from commercial groups and bots in their analysis. As part of the preparations and data collection for our study, we found that 75.51% (52,301/69,264) of psoriasis tweets in English sent between February 2016 and October 2018 by users in the United States were commercial or bot-like in nature. To our knowledge, there are no additional studies that have used Twitter to gain a more profound understanding of patients’ attitudes toward seeking psoriasis treatment.

### Study Objective and Research Questions

The objective of this study is to conduct a content analysis of Twitter posts (in English) published by users in the United States between February 1, 2016, to October 31, 2018, to examine perspectives that potentially influence the treatment decision among patients with psoriasis. We intend to answer the following research questions:

1. What perspectives toward seeking treatment are being expressed by psoriasis patients on Twitter?
2. What are the demographics (ie, gender, race/ethnicity) of these psoriasis patients on Twitter?
3. What is the volume of unique Twitter users who talk about this topic?
4. What are the predominant themes in the conversations among psoriasis patients?
5. For commercial and bot-like tweets, what types of treatments are being promoted?

This protocol paper provides a detailed description of a social media research project including the process of data extraction, cleaning, and analysis. It is our goal to contribute to the development of more transparent social media research efforts. Our findings will shed light on whether Twitter provides a promising data source for garnering patient perspective data about psoriasis treatment decisions. The data will also help to determine whether Twitter might serve as a potential outreach platform for raising awareness of psoriasis and treatment options among patients and for implementing related health interventions.

## Methods

### Study Type

This is a qualitative study that will analyze user-generated posts about psoriasis from the social network Twitter.

### Data Source

Twitter posts in English containing terms related to psoriasis will be obtained for the time period from February 1, 2016, to October 31, 2018. To access public Twitter user data, we will use Symplur Signals [49], a health care social media analytics company that maintains the largest publicly available database of health care and disease-related conversations with the globally recognized Healthcare Hashtag Project. Symplur Signals extracts data from the Twitter REST API (representational state transfer application programming interface) and makes it available to researchers; it is commonly used in peer-reviewed research [50-54]. Symplur Signals data are updated daily and easily sortable by social media user type (eg, patient, physician, health care organization), location and time zone, language, disease/health interests, and Twitter message content. The location of the users (limited to users within the United States) will be determined using a mapped location filter as defined by Gnip Inc, a social media data provider, and based on the Profile Geo 2.0 algorithm [55]. That algorithm uses a number of data points to determine a user’s location including the self-reported bio location in the Twitter user profile and geotracking data if available. We extracted data from Twitter through the Symplur Signals user interface, searching for the keyword and hashtags listed in Multimedia Appendix 1. The data were provided in an Excel (Microsoft Corp) file, which we further analyzed on local university computers.

### Search Filters

We will use a framework for data collection, quality assessment, and reporting standards as well as for developing search filters for social media data as previously suggested by Kim et al [56]. The root terms we will use to collect the sample of tweets are listed in Multimedia Appendix 1. These terms can appear in the post or in an accompanying hashtag, for example, “psoriasis” or #PsoriasisChat. We will select keywords and hashtags based on expert knowledge (clinicians, social media experts) and use a systematic search of topic-related language based on data in Symplur Signals.

### Data Cleaning and Debiasing

The following types of irrelevant tweets will be excluded: retweets (ie, messages shared by Twitter users that other users composed) and non-English language tweets identified using the Liu method. Liu et al [57] developed and evaluated a web-based language identification tool called langid.py that uses natural language processing techniques and assists with text categorization in specific languages. They showed that the tool maintains consistently high accuracy. Furthermore, we will use the program Botometer (formerly BotOrNot) to identify Twitter accounts by social bots or commercial groups that could possibly influence the results and introduce bias [58,59]. Automated accounts on Twitter created by industry groups and private companies promote specific ideas or products and, thus, influence discussions. Botometer is a publicly available service launched in 2014 and includes more than 1000 variables to assess the extent to which a Twitter account exhibits characteristics of social bots [60]. Variables include the account network (ie, diffusion patterns), user data (ie, metadata), friends (ie, account’s contacts), tweet rate, and sentiment and content of the account messages. The classification system generates a score that determines the likelihood of any one account being a social bot. Davis et al [60] demonstrated that the program scores a detection accuracy above 95%. If an account is identified as a social bot, that account and any tweets produced from that account will be removed from our dataset so we can focus on analyzing patient’s perspectives.

### Data Privacy and Confidentiality

All analyses will adhere to the terms and conditions, terms of use, and privacy policies of Twitter. We will further abide by University of Southern California (USC) institutional review board (IRB) regulations and the USC Privacy of Personal Information policy.

All data will be entered into a computer and database that is password protected. The study data will be collected using the system Research Electronic Data Capture at USC, which is a secure, web-based app designed to support data capture for research studies. Provision of data to the IRB, National Institutes of Health (NIH), and FDA is facilitated by this database system.

Any identifying and personal health information will be redacted from the dataset by the coders. Information that might identify a contributor’s identity will be redacted from any report developed to share the findings, and any Twitter posts we include in publications will be paraphrased to protect the privacy of the users.

### Data Analysis

We will use a standard coding approach for characterizing the Twitter messages and users. Two independent team members will be responsible for coding based on a set of a priori classifiers listed in Multimedia Appendix 2 and 3. Information available in a user’s Twitter profile (ie, username, description, avatar image) will be used to characterize the user of the Twitter account who generated the post to determine if the individual is a psoriasis patient (Multimedia Appendix 3). In other words, we will characterize a Twitter user as a psoriasis patient if they specifically mention being a patient in their description or previous tweets. We will further code the person’s gender and race/ethnicity (White person versus person of color) if the Twitter profile contains sufficient information to do so.

We will then code the Twitter messages from psoriasis patients (Multimedia Appendix 2). Individual Twitter posts will be classified as posts originating from these patients either if the user who authored the message was already classified as a psoriasis patient through examination of their Twitter profile or if the post mentioned psoriasis in the first person (eg, “Haven’t felt myself lately. Asked my doc about an alternative treatment plan today.”) We will analyze the messages from these patients to identify the health issues and concerns they express (Multimedia Appendix 2).

Cohen kappa will be calculated for each code category to assess interrater reliability [61,62]. Once we establish concordance in the coder’s classification with Cohen kappa greater than .80 for each coding category, the remaining data will be divided between the two coders. The project principal investigators will help to establish consensus in instances where coders disagree.

### Statistical Analysis

This study will rely on public, anonymized data and adhere to the terms and conditions, terms of use, and privacy policies of Twitter. The proposed work received IRB approval from the authors’ university.

We will use descriptive statistics to analyze the data and identify the most prevalent topics in the Twitter content. Units of analysis will be unique terms in posts as well as the number of Twitter messages and users (ie, patients). We will describe the patient characteristics focusing on gender and race/ethnicity, as displayed on Twitter. For each tweet theme analysis, we will present findings in a confusion matrix where the diagonal line indicates the prevalence of a topic and the off-diagonal lines indicate topic overlap. The number of posts containing 2 or more topics would be found at the intersection of the matrix for these topics. Representative examples of tweets within each category will be selected to illustrate additional themes and will be shown as paraphrased quotes to protect users’ privacy.

### Risk Analysis

The described work presents minimal risk research. We will use public user data from the social network Twitter. Patient identifiers do not apply. Identifiable information such as human subjects’ names and Twitter handles will not be included in the analysis dataset.

### Dissemination of Study Findings

The authors plan to publish the study findings in a peer-reviewed journal and at topic-related conferences (to be determined at a later date). All listed authors and/or contributors are compliant with guidelines outlined by the International Committee of Medical Journal Editors for author inclusion in a published work.

## Results

Study approval was obtained from the IRB at USC (protocol HS-18-00867). Data extraction and cleaning are complete. For the time period from February 1, 2016, to October 31, 2018, we obtained 95,040 Twitter posts containing terms related to psoriasis from users in the United States published in English. After removing duplicates, retweets, and non-English tweets, we found that 75.51% (52,301/69,264) of the psoriasis-related posts were sent by commercial or bot-like accounts, while 16,963 posts were noncommercial and will be included in the analysis to determine the patient perspective (see Multimedia Appendix 4 for detailed data extraction and cleaning flow diagram). Analysis was completed in Summer 2020.

## Discussion

### Limitations

This exploratory pilot study is limited to Twitter conversations from people who use words and hashtags related to psoriasis in their Twitter posts. As a result, we will only include those patients’ posts in the dataset who are familiar with the term “psoriasis” and not include posts from patients who might talk about their disease experience on Twitter but don’t include any of these words.

The generalizability of the study is somewhat limited, because Twitter messages from locations outside of the United States and messages in other, non-English languages will not be included. We also recognize that this type of social media research favors those with internet access and could, therefore, lead to potential bias in the research data. Twitter users tend to be younger (38% are aged 18 to 29 years), college graduates (32%), and located in urban areas (26%) [17].

### Practical Significance

If successful, our findings will shed light on whether Twitter provides a promising data source for garnering patients’ perspectives about psoriasis treatment decisions. The data will also help to determine whether Twitter might serve as a potential outreach platform for raising awareness of psoriasis and treatment options among patients and implementing related health interventions. This protocol paper provides a detailed description of a social media research project including the process of data extraction, cleaning, and analysis. It is our goal to contribute to the development of more transparent social media research efforts.

## Appendix

Multimedia Appendix 1 Keywords and hashtags to assess attitudes toward treatment among patients with psoriasis on Twitter. The selection is based on data from Symplur Signals.

Multimedia Appendix 2 Code categories to identify main themes in Twitter posts related to psoriasis.

Multimedia Appendix 3 Code categories to classify Twitter users.

Multimedia Appendix 4 Data extraction and cleaning flow diagram.

## Abbreviations

FDA: US Food and Drug Administration
IRB: institutional review board
NIH: National Institutes of Health
PGHD: patient-generated health data
REST API: representational state transfer application programming interface
USC: University of Southern California
