# Supplementary material for: Perspectives Toward Seeking Treatment Among Patients With Psoriasis: Protocol for a Twitter Content Analysis
Source: JMIR Res Protoc. 2021 Feb 18;10(2):e13731. doi: 10.2196/13731 (PMC7932841; doi:10.2196/13731)
Supplement: Multimedia Appendix 1 [file resprot_v10i2e13731_app1.pdf]

Multimedia Appendix 1. Keywords and hashtags to assess attitudes toward treatment among patients with psoriasis on Twitter. The selection is based on data from Symplur Signals.

Topic	Keywords	Hashtags
**Psoriasis**	Psoriasis	#psoriasis<br>#discoverpsoriasis<br>#WorldPsoriasisDay<br>#PsoriasisChat<br><br>***Exclude:***<br>Psoriasis-related: #psoriaticarthritis, #Parapsoriasis<br><br>Spanish and other languages: #psoriasi, #psoriasismásallá, #defiendetupsoriasis, #aclaralapsoriasis, #entdeckepsoriasis, #DecouvrirLePsoriasis, #DecouvrezLePsoriasis, #LaPsoriasisQueNoSeVe, #psoriasismasalla, #psoriasisinfantil<br><br>Non-active: #discoverpsoriasis, #entdeckepsoriasis, #Psoriasis360, #Psoriasis2016, #congresopsoriasis, #PustularPsoriasis, #deseosxpsoriasis, #besosporlapsoriasis, #LaPsoriasisQueNoSeVe, #congresopsoriasis
