# Supplementary material for: Perspectives Toward Seeking Treatment Among Patients With Psoriasis: Protocol for a Twitter Content Analysis
Source: JMIR Res Protoc. 2021 Feb 18;10(2):e13731. doi: 10.2196/13731 (PMC7932841; doi:10.2196/13731)
Supplement: Multimedia Appendix 2 [file resprot_v10i2e13731_app2.pdf]

Multimedia Appendix 2. Code categories to identify main themes in Twitter posts related to psoriasis.

Code category	A priori codes/variables and definitions (data dictionary code value)	Emergent codes/variables and definitions (code value)
Attitudes toward seeking treatment	• Seeking treatment (defined as: person seems to be inclined to see/is interested in seeing a doctor)<br>• Not seeking treatment (defined as: person does not seem to be interested in seeing a doctor)<br>• Unclear<br>• Not applicable e.g. business account	
Reasons for not seeking treatment	• Health literacy (defined as: awareness of treatment options)<br>• Access to care/healthcare utilization (defined as: having access to health care, or the lack thereof)<br>• Insurance (defined as: having insurance, or the lack thereof)<br>• Cost (defined as: cost may be too high)<br>• Dissatisfaction with treatment (defined as: person is not satisfied with treatment and does not seek further treatment)<br>• Side-effects (defined as: an unacceptable complication of treatment)<br>• Unclear<br>• Not applicable e.g. business account	
Reasons for seeking treatment	• Desire to change treatment (defined as so. Is seeking to try a different treatment)<br>• Quality of life : the individual's' perception of their position in life, in the context of the cultural and value systems in which they live and in relation to their goals, expectations, standards and concerns per WHO<br>• Unclear<br>• Not applicable e.g. business account	

<table>
<tr><td>Treatment mentioned and/or promoted (data will be stratified by patients vs. healthcare provider vs. other marketing)</td><td>Topicals (defined as: creams, ointments, oils, solutions, anything that is put directly on the skin)
Pills/Drugs (defined as: medication taken by mouth)
Injectable medications (defined as: regular injections of medications used to control psoriasis)
Phototherapy (defined as: UVB lamp, lightbox)
Infusions (defined as: undergoing IV infusions of medications)
Alternative medicine
Laser therapy
Nutrition
Unclear, e.g., medication or treatment mentioned, but unclear what kind
Not applicable - no treatment mentioned</td><td></td></tr>
<tr><td colspan="3">Medications</td></tr>
<tr><td></td><td>Topical treatment (defined as: treatment mentioned and/or promoted)
<u>Topical treatment includes</u>:

Clobetasol propionate, Augmented betamethasone, Clobetasol propionate
Desonide, Desoximetasone, Diflorasone diacetate, Fluocinolone acetonide, Fluocinonide, Flurandrenolide, Fluticasone propionate, Hydrocortisone, Hydrocortisone valerate, Mometasone furoate, Alclometasone dipropionate, Betamethasone valerate, Coal Tar, Desonide, Desoximetasone, Dovonex (calcipotriene), Flurandrenolide, Halcinonide, Halobetasol propionate, Hydrocortisone butyrate, Hydrocortisone probutate, Prednicarbate, Salicylic Acid, Taclonex (calcipotriene and betamethasone dipropionate), Tazorec (tazarotene), Triamcinolone acetonide, Vectical (calcitriol), Zithranol-RR (anthralin)</td><td></td></tr>
<tr><td></td><td>Biologic Drugs
<u>Biologic drugs include</u>:

Cimzia (certolizumab pegol)
Enbrel (etanercept)
Humira (adalimumab)
Remicade (infliximab)
Stelara (ustekinumab)
Cosentyx (secukinumab)
Taltz (ixekizumab)</td><td></td></tr>
</table>

	Siliq (brodalumab)<br>Tremfya (guselkumab)<br>Ilumya (tildrakizumab-asmn)<br>Amjevita (adalimumab-atto)<br>Cyltezo (adalimumab-adbm)<br>Erelzi (etanercept-szzs)<br>Inflectra (infliximab-dyyb)<br>Renflexis (infliximab-abda)	
	• Oral Treatments<br><br>Oral Treatments include:<br><br>Cyclosporine<br>Methotrexate<br>Soriatane (Acitretin)<br>Hydrea (hydroxyurea)<br>Isotretinoin<br>Mycophenolate mofetil<br>Sulfasalazine<br>6-thioguanine<br>Otezla (apremilast)<br>Xeljanz and Xeljanz XR (tofacitinib)	
