# Supplementary material for: Perspectives Toward Seeking Treatment Among Patients With Psoriasis: Protocol for a Twitter Content Analysis
Source: JMIR Res Protoc. 2021 Feb 18;10(2):e13731. doi: 10.2196/13731 (PMC7932841; doi:10.2196/13731)
Supplement: Multimedia Appendix 3 [file resprot_v10i2e13731_app3.pdf]

## Multimedia Appendix 3. Code categories to classify Twitter users.

| Code category | A priori codes/variables and definitions (data dictionary code value) | Emergent codes/variables and definitions (code value) |
|---|---|---|
| Individual/Entity | Individual<br>• Patient with psoriasis<br>• Healthcare professional<br>• Healthcare activist<br>• Healthcare provider<br>• Healthcare educator/coach<br>• Other – individual<br>Institution/Group<br>• Institutional healthcare provider<br>• Advocacy group<br>• Other – group/organization<br><br>• Unclear<br>• Data not available | |
| Sex | • Female<br>• Male<br>• Transgender (Male, Female<br>• Other (e.g., Queer)<br>• Unclear<br>• Data not available<br>• Not applicable | |
| Race | • White or Caucasian<br>• Person of color<br>• Unclear<br>• Data not available<br>• Not applicable | |
| Ethnicity | • Latino/Hispanic<br>• Not Latino/Not Hispanic<br>• Unclear<br>• Data not available<br>• Not applicable | |
