# Supplementary figures and images for: Perspectives Toward Seeking Treatment Among Patients With Psoriasis: Protocol for a Twitter Content Analysis
Source: JMIR Res Protoc. 2021 Feb 18;10(2):e13731. doi: 10.2196/13731 (PMC7932841; doi:10.2196/13731)

**Multimedia Appendix 4. Data extraction and cleaning flow diagram.**

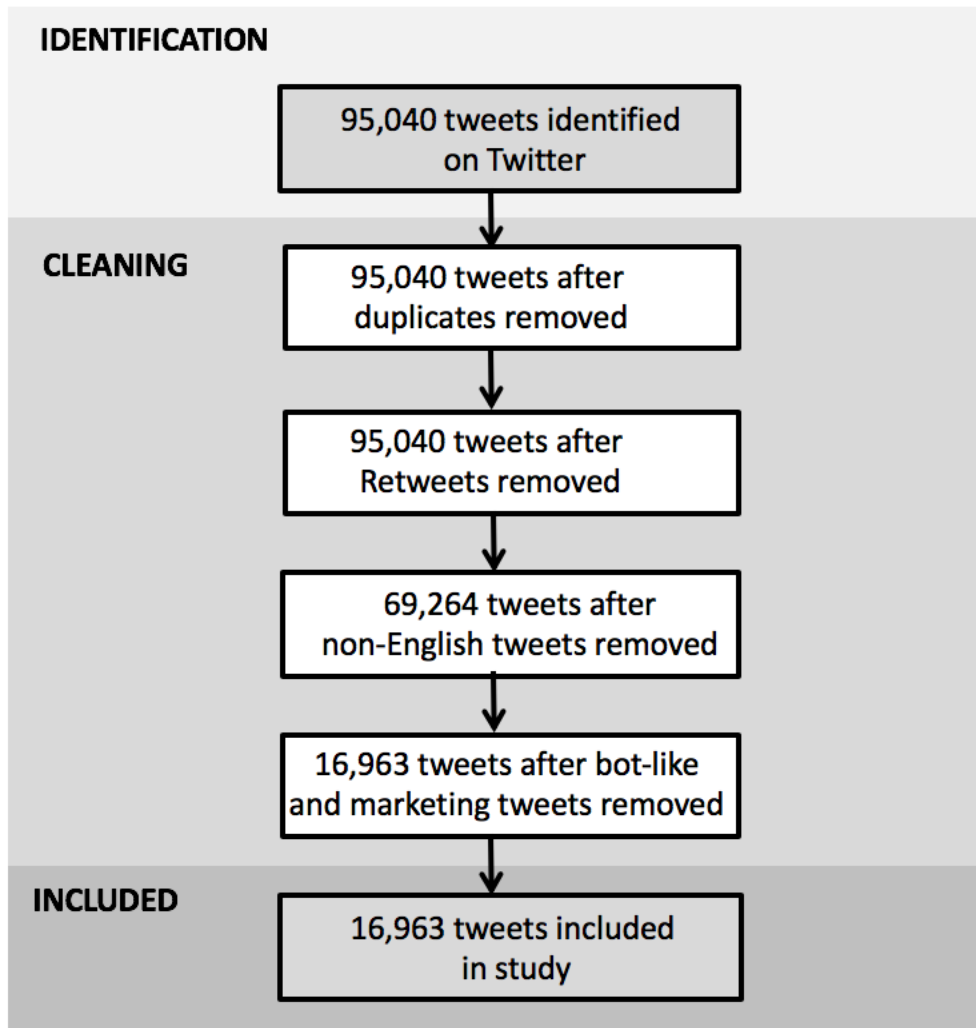

Supplement: Multimedia Appendix 4 [file resprot_v10i2e13731_app4.pdf]
